# O6.8. GLUTAMATERGIC DYSFUCTION AND TREATMENT RESPONSE IN MINIMALLY TREATED AND CHRONIC SCHIZOPHRENIA PATIENTS

**DOI:** 10.1093/schbul/sby015.229

**Published:** 2018-04-01

**Authors:** Elias Mouchlianitis, Lucy Vanes, Erica Barry, Krisna Patel, Katie Wong, Lilla Porfy, Sukhi Shergill

**Affiliations:** Institute of Psychiatry, Psychology and Neuroscience, King’s College London

## Abstract

**Background:**

Glutamatergic dysfunction as a result of NMDA receptor hypofunction has been implicated in antipsychotic treatment-resistant schizophrenia, however its nature in very early stages and chronic stages of the disease is still unknown. Data on glutamate and treatment response are currently limited in two separate studies, one in first-episode patients (Egerton et al., 2012) and one in chronic patients (Mouchlianitis et al., 2016). Here we acquired proton magnetic resonance spectroscopy measures from a large sample of minimally treated first episode and chronic schizophrenia patients, and a group of matched healthy controls. Both first-episode and chronic schizophrenia groups were further stratified by treatment response. This allowed us to investigate glutamatergic dysfunction in both early and later stages of the diseases in relation to treatment-response.

**Methods:**

We acquired proton magnetic resonance spectroscopy (1H-MRS) at 3 Tesla from bilateral anterior cingulate cortex (ACC) from 170 participants. 137 participants with a diagnosis of schizophrenia (according to ICD-10 criteria) and 33 healthy controls matched for age, sex, and socioeconomic background consented to participate in this study. The patient sample included 95 minimally treated first-episode patients, with illness duration less than 36 months, of which 65 has shown good response and 26 have shown persistent psychotic symptoms; and a group of 42 chronically-ill patients with illness duration over 3 years. The chronic group was classified into 21 antipsychotic treatment-resistant patients and 21 antipsychotic treatment-responsive patients. 1H-MRS data were analyzed using a standard basis function within LC-Model. Our primary measure was glutamate to creatine ratio (Glu/Cre) and its correlation to N-Acetylaspartic acid to creatine ratio (NAA/Cre).

**Results:**

The main new finding is that first-episode patients with persistent psychotic symptoms show significantly higher Glu/Cr and NAA/Cr correlation R(23)=0.76, P<0.001.compared to first-episode patients in remission R(65)=0.43, P<0.00, Fisher’s r-to-z, Z=1.97, P<0.05, effect size d=0.48. Compared to healthy controls (who did not show any Glu/Cr to and NAA/Cr correlation R(33)=0.24, P=0.33) the FEP-resistant group showed a significant difference, Z=2.6, P<0.005, representing a large effect size of d=0.87 but not the FEP-responsive group, Z=0.97, P=0.17. Remarkably, when we examined first-episode patients with antipsychotic exposure of less than 6 months, we found an extremely high correlation in the non-responsive group R(5)=0.95, P=0.01, compared to the responsive-group,R(20)=0.44, P<0.05, which reflected a large effect size of d=0.99. Chronically-ill resistant patients showed a significant correlation R(21)=0.48, P<0.05 and responsive trend-level correlation R(21)=0.41, P=0.07, but neither group differed from healthy controls.

**Discussion:**

Our study provides the first 1H-MRS evidence for acute metabolic perturbations in glutamatergic neurotransmission in minimally treated schizophrenia patients with persistent psychotic symptoms. These are absent in later stages of the disease for both treatment-resistant and treatment-responsive patients. It is likely that neurodegenerative processes resulting from excitotoxity due glutamatergic dysfuntion are most impactful within the first few months from illness onset. Our data point to the urgent need to identify reliable biomarkers for the prediction of antipsychotic treatment-response and the development of novel interventions to address glutamatergic perturbations at the beginning of their illness.

